# Polyphosphatase PPN1 of *Saccharomyces cerevisiae*: Switching of Exopolyphosphatase and Endopolyphosphatase Activities

**DOI:** 10.1371/journal.pone.0119594

**Published:** 2015-03-05

**Authors:** Nadezhda Andreeva, Ludmila Trilisenko, Mikhail Eldarov, Tatiana Kulakovskaya

**Affiliations:** 1 Skryabin Institute of Biochemistry and Physiology of Microorganisms, Russian Academy of Sciences, pr. Nauki 5, Pushchino, 142290, Russia; 2 Centre “Bioengineering”, Russian Academy of Sciences, pr. Shestidesyatiletiya Oktyabrya 7–1, Moscow, 117312, Russia; Louisiana State University Health Sciences Center, UNITED STATES

## Abstract

The polyphosphatase PPN1 of *Saccharomyces cerevisiae* shows an exopolyphosphatase activity splitting phosphate from chain end and an endopolyphosphatase activity fragmenting high molecular inorganic polyphosphates into shorter polymers. We revealed the compounds switching these activities of PPN1. Phosphate release and fragmentation of high molecular polyphosphate prevailed in the presence of Co^2+^ and Mg^2+^, respectively. Phosphate release and polyphosphate chain shortening in the presence of Co^2+^ were inhibited by ADP but not affected by ATP and argininе. The polyphosphate chain shortening in the presence of Mg^2+^ was activated by ADP and arginine but inhibited by ATP.

## Introduction

Inorganic polyphosphate (PolyP), the linear polymer containing a few to several hundred phosphate residues, performs many functions in living cells. PolyP participates in the regulation of gene expression, stress response and virulence in bacterial cells [1–4]. In human organism, PolyP is involved in the regulation of Ca^2+^ uptake in mitochondria [5], bone tissue development [6–8], blood coagulation [9], and brain functions [10].

Probably, there is a relationship between the multiple functions of PolyP and the ability of enzymes of its metabolism to catalyze the conversion of other substrates. This ability is characteristic of many PolyP-depending enzymes. Polyphosphate-glucose phosphotransferase uses both PolyP and ATP as phosphoryl donors [11]. The gppA exopolyphosphatase splits P_i_ from PolyP and bacterial second messengers: guanosine 5’-triphosphate, 3’-diphosphate and guanosine 5’-diphosphate, 3’-diphosphate [12]. Some bacterial exopolyphosphatases possess substantial nucleoside triphosphatase activities [13]. The enzymes belonging to the polyphosphate kinase 2 subfamily catalyze nucleoside monophosphate phosphorylation [14]. The yeast vacuolar membrane chaperon Vtc4 is a polyphosphate synthetase [15]. The *S*. *cerevisiae* protein DDP1 (a diadenosine and diphosphoinositol polyphosphate phosphohydrolase) catalyzes the fragmentation of high molecular PolyP into shorter polymers [16]. The exopolyphosphatase PPX1 of *S*. *cerevisiae* hydrolyses adenosine tetraphosphate and guanosine tetraphosphate to ATP and GTP, respectively [17].

The polyphosphatase PPN1 is an important enzyme of PolyP metabolism in yeasts (http://www.uniprot.org/uniprot/Q04119). The increase in PolyP content and chain length in the cytoplasm and mitochondria is observed in the *ΔPPN1* mutant [18]. The data on the reactions catalyzed by this enzyme are contradictory. Previously, PPN1 was defined as an endopolyphosphatase (polyphosphate depolymerase, EC 3.6.1.10) fragmenting the long chained PolyP into shorter ones [19, 20]. The exopolyphosphatase (polyphosphate phosphohydrolase EC 3.6.1.11) activity of PPN1 splitting orthophosphate (P_i_) from the polymer chain end was shown later [20, 21].

The goal of this study was to reveal the effectors that can switch the exopolyphosphatase and endopolyphosphatase activities of *S*. *cerevisiae* PPN1.

## Materials and Methods

### Chemicals

The reagents used in the work were Yeast Nitrogen Base (Difco, USA); GF-A filter (Whatmann, United Kingdom); Butyl-Toyopearl 650M (Toson, JP); PolyP_208_ (inorganic polyphosphate with the average chain length of 208 phosphate residues) (Monsanto, USA). All other chemicals were obtained from Sigma–Aldrich.

### Purification of polyphosphatase PPN1

The strain CRN/pMB1_PPN1 Sc of *S*. *cerevisiae* overexpressing the polyphosphatase PPN1 [22] was maintained on an agarized YNB medium. The strain was grown at 29°C in flasks with 200 mL of YNB at 120 rpm for 24 h to obtain biomass. The YNB medium contained (g/L): Yeast Nitrogen Base, 1.7; glucose, 20; L-tryptophane, L-histidine, L-methionine, adenine, 0.02; L-leucine, 0.06. The biomass was harvested by centrifugation at 3000 g and washed twice with cold distilled water. Spheroplasts were obtained as described [22] and treated at 0°C in a glass homogenizer with a Teflon pestle in 25 mM Tris-HCl (pH 7.2) containing 0.5 mM phenylmethylsulphonyl fluoride. The lysate was centrifuged at 5000 g 10 min. The precipitate was treated again under the same conditions and centrifuged. The supernatants were combined, centrifuged at 13000 g for 60 min, and used for enzyme purification. The purification procedure was performed at 0°C. Ammonium sulfate was added to the cellular extract up to 50% saturation, and the precipitate was obtained in 1 h by 20-min centrifugation at 12000 g. The supernatant was filtered through a GF-A filter and put on Butyl-Toyopearl 650M resin equilibrated with 50 mM Tris-HCl, pH 7.2, with 50% ammonium sulfate. In 45 min, the resin was precipitated at 4000 g for 3 min and washed three times with 50 mM Tris-HCl, pH 7.2, containing 50% ammonium sulfate. For polyphosphatase elution, the resin was washed four times with 50 mM Tris-HCl, pH 7.2, containing 25% ammonium sulfate. Triton X-100 (0.05%) was added to the resultant preparation. After ultrafiltration through an YM-10 membrane, the preparation was applied to Heparin-agarose equilibrated with 25 mM Tris-HCl, pH 7.2, containing 0.1% Triton X-100. In 2 h, the resin was precipitated and washed twice with 25 mM Tris-HCl, pH 7.2, containing 0.1% Triton X-100; then it was washed seven times with the same buffer containing 0.7 M KCl. The polyphosphatase was eluted with 25 mM Tris-HCl, pH 7.2, containing 0.1% Triton X-100 and 1 M KCl.

The preparation was stored at-20°C. The purity of polyphosphatase was assayed by electrophoresis in 12.5% polyacrylamide gel in the presence of SDS.

### Enzyme activity assay

Exopolyphosphatase and endopolyphosphatase activities were assayed at 30°C. Exopolyphosphatase activity was assayed by the rate of P_i_ formation [22]. The amount of the enzyme forming 1 μmole of P_i_ per 1 min was taken as a unit of enzyme activity (U). The incubation medium contained: Tris-HCl, 50 mM (pH 7.2); NH_4_Cl, 200 mM; PolyP_208_ (as P_i_), 2.5 mM. The medium also contained CoSO_4_ (0.1 mM) or MgSO_4_ (0.25 mM) as indicated in the figure and table legends. In some cases, 9.2 mM PolyP_208_ was used.

Endopolyphosphatase activity was estimated by the chain length of hydrolysis products [19, 20] using PolyP_208_ as a substrate. The incubation medium (1 ml) contained Tris-HCl, 50 mM (pH 7.2); NH_4_Cl, 200 mM; PolyP_208_, 9.2 mM; CoSO_4_, 0.1 mM, or MgSO_4_, 0.25 mM; and 23 mU of the enzyme (as exopolyphosphatase). The reaction was stopped by the addition of 5.8 μl 60% HClO_4_ to 0.1 ml samples, which were taken from the incubation medium. After 3-min cooling, 6 M NaOH (8.3 μl) and an equal volume of glycerol were added to the samples. The samples (20 μl) were subjected to PAGE in 24% polyacrylamide gel with 7 M urea [19, 20]. The gels were stained with 0.05% toluidine blue in a water solution containing 25% methanol and 1% glycerol and then washed with distilled water [19]. Commercial PolyP with the average chain lengths of 15, 25, 75 and 208 phosphate residues were used as markers.

The pyrophosphatase, ATPase and unspecific phosphatase activities were assayed as described earlier [23]. Protein was assayed according to [24] with BSA as a standard.

## Results

**Fig 1:**
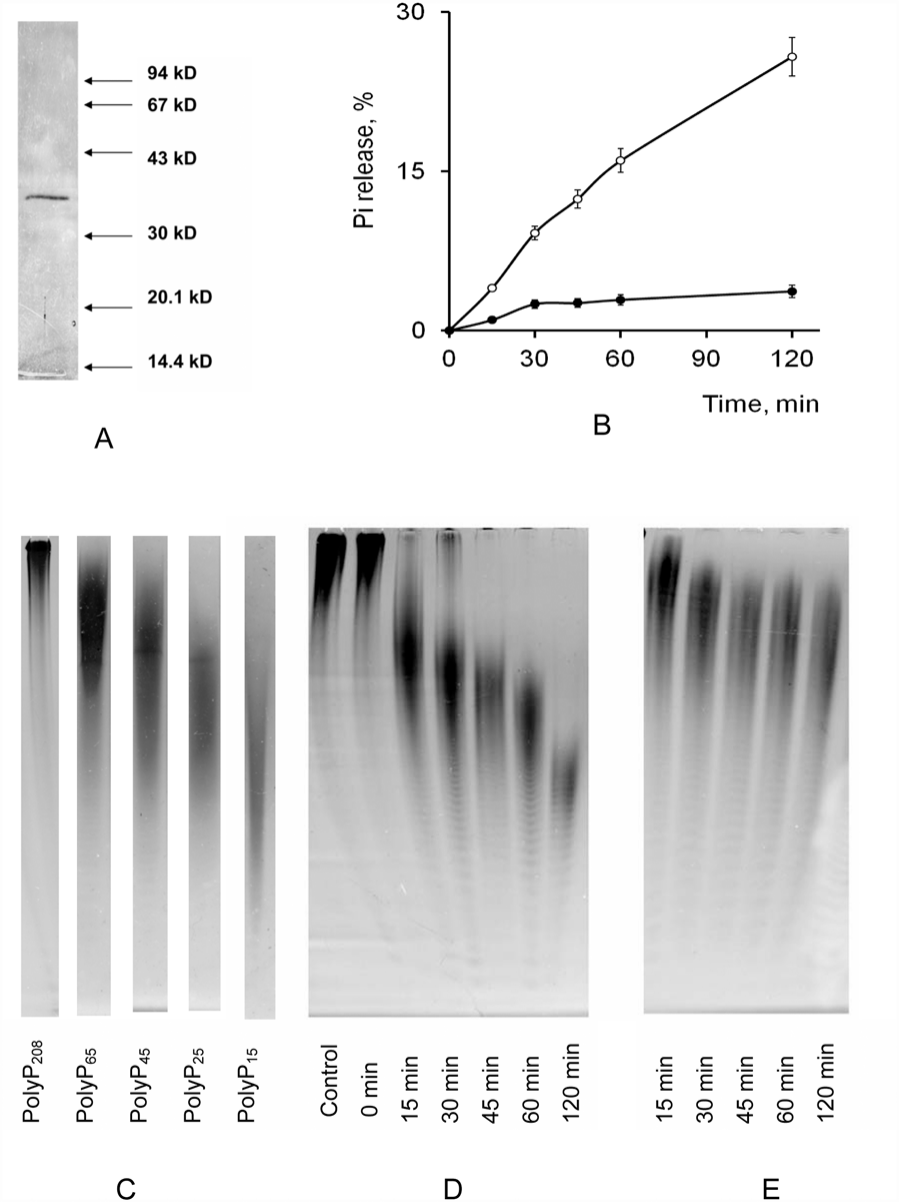
The SDS-PAGE of purified protein (A) and time dependence of PolyP_208_ hydrolysis by the purified PPN1 (B-E). The protein markers (A) were phosphorylase b (94 kD), albumin (67 kD), ovalbumin (43 kD), carbonic anhydrase (30 kD), trypsin inhibitor (20.1 kD), α-lactalbumin (14.4 kD). B, P_i_ release, % of P in PolyP_208_ used as substrate; (○) in the presence of 0.1 mM CoSO_4_, (●) in the presence of 0.25 mM of MgSO_4_. C-E, PolyP PAGE was performed in 24% polyacrylamide gel with 7M urea; toluidine blue staining. C, PolyP markers, commercial PolyP with the average chain lengths of 15, 25, 45 and 65 phosphate residues from Sigma (USA) and PolyP with an average chain length of 208 phosphate residues from Monsanto (USA). D, hydrolysis products of PolyP_208_ in the presence of 0.1 mM CoSO_4_. E, hydrolysis products of PolyP_208_ in the presence 0.25 mM of MgSO_4_. Control, PolyP_208_ was incubated without the enzyme at 30°C for 120 min. The experiments were repeated in triple and the average values and typical photograph are shown.

The purified recombinant PPN1 was obtained from the cells of the strain CRN/pMB1_PPN1 Sc (Table 1). PAGE showed one polypeptide band with a molecular mass of ~33 kDa (Fig. 1A). The PPN1 gene encodes a polypeptide with the predicted molecular mass of 78 kDa, whereas the monomer molecular mass of the mature PPN1 purified from wild yeast strains is about 33–35 kDa [20–21]. PPN1 undergoes specific proteolytic maturation [19]. The tryptic digest peptides of recombinant PPN1 preparations from the above strain were the same as the peptides of the mature wild PPN1 [25]. The enzyme had no activity with pyrophosphate (PP_i_), ATP and p-nitrophenylphosphate (data not shown). The specific exopolyphosphatase activity in the presence of 0.1 mM CoSO_4_ was 290, 240, and 38 U/mg of protein with PolyP_208_, PolyP_15_, and tripolyphosphate (PolyP_3_), respectively. Thus, the recombinant PPN1 was similar to the wild type enzyme in molecular mass and substrate specificity [21].

**Table 1:** Purification of recombinant PPN1 of *S. cerevisiae*.

Purification stage	Protein, mg	Total activity, U	Specific activity, U/mg protein	Yield, %
**Cellular extract**	101±7.2	408±4.0	4.2	100
**Ammonium sulfate (50% of saturation), supernatant**	37.5±5.0	300±3.5	8.0	74
**Butyl-Toyopearl 650M**	10±0.4	170±2.7	17	42
**Heparin-agarose**	0.14±0.05	40±0.5	290	9.8

The enzyme activity at each purification step was measured by P_i_ release in the medium containing 50 mM Tris-HCl, pH 7.2, 200 mM of NH_4_Cl, 2.5 mM PolyP_208_ (as P_i_), and 0.1 mM CoSO_4_. The activity and protein assays were repeated in triple and the average value with standard deviations are given.

**Fig 2:**
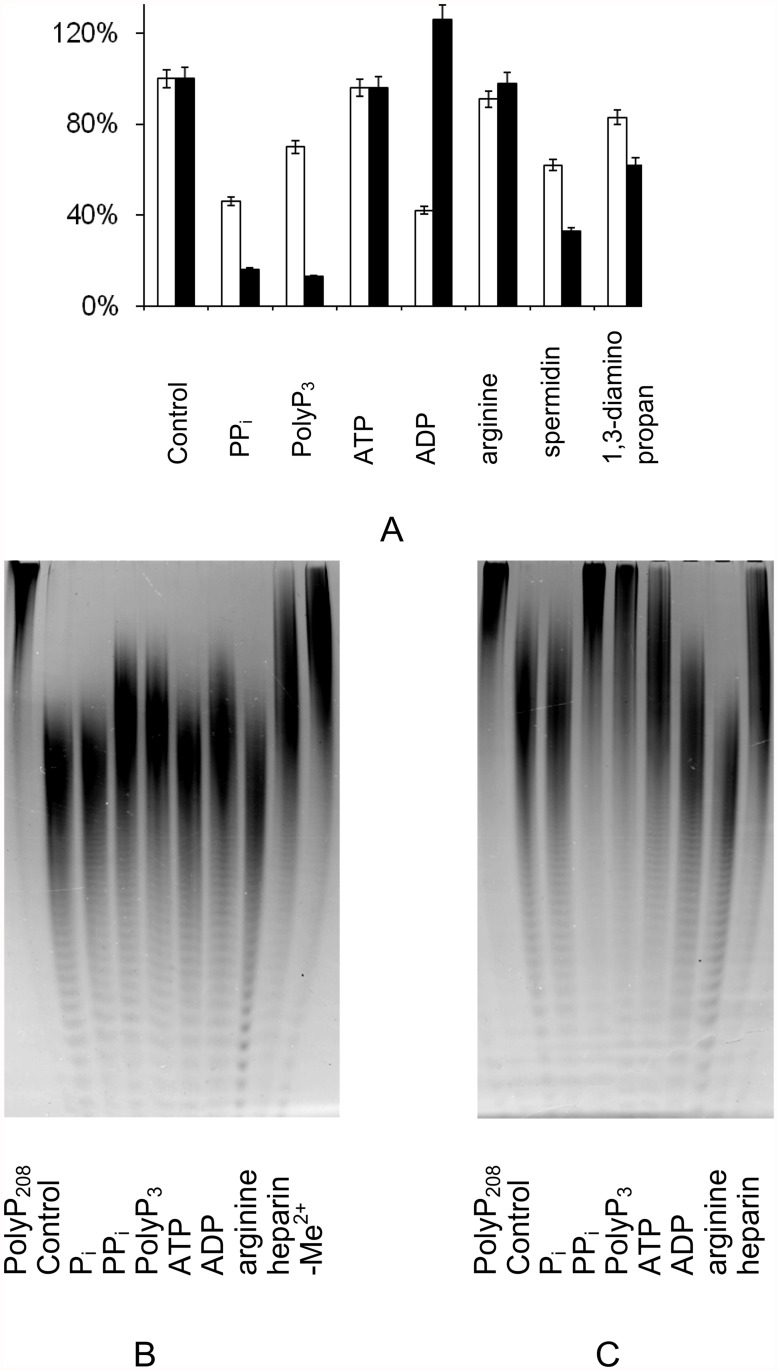
The effects of some compounds on exopolyphosphatase (A) and endopolyphosphatase (B, C) activities of purified PPN1 with PolyP_208_ in the presence of 0.1 mM Co^2+^ or 0.25 mM Mg^2+^. The concentrations of effectors are (mM): P_i_, PP_i_, PolyP_3_, ATP and ADP, 2.0; arginine, 100; spermidin and 1,3-diaminopropan, 50. The concentration of heparin was 0.01 mg/ml. Control, without effectors. A, white columns – exopolyphosphatase activity in the presence of Co^2+^, 100% corresponds to 290 U/ mg protein; black columns – exopolyphosphatase activity in the presence Mg^2+^, 100% corresponds to 22 U/mg protein. B, the endopolyphosphatase activity in the presence of Co^2+^; C, the endopolyphosphatase activity in the presence of Mg^2+^; - Me^2+^, without bivalent metal cation. The reaction time was 60 min. The PolyP PAGE was performed in 24% polyacrylamide gel with 7M urea; toluidine blue staining. PolyP_208_, the substrate was incubated without the enzyme for 60 min. The experiments were repeated in triple and the average values and typical photographs are shown.

The exopolyphosphatase activity of PPN1 is very low without bivalent cations [25, 26]. The maximal exopolyphosphatase activity of recombinant PPN1 was observed in the presence of 0.1 mM Co^2+^ ions [25]. The optimal concentration of Mg^2+^ was 0.25 mM; however, the exopolyphosphatase activity with this cation was several times lower than with Co^2+^ [25]. These values are similar to the value for the wild type enzyme [26]. There are no data on the effects of these cations on the endopolyphosphatase activity of PPN1. We have compared the time dependence of P_i_ release (Fig. 1B) and PolyP chain shortening (Fig. 1C-E) in the presence of Co^2+^ or Mg^2+^. The enzyme effectively released P_i_ in the presence of Co^2+^ (Fig. 1B). Simultaneously, the chain length of PolyP decreased from ~208 to ~15 phosphate residues (Fig. 1D). P_i_ release was insignificant in the presence of Mg^2+^ (Fig. 1B), though the chain length of the substrate decreased from ~208 to ~45–60 phosphate residues already in the first 30 min of the reaction and then remained the same (Fig. 1E). Probably, the PolyP_45–60_ is not a suitable substrate for PPN1 in the presence of Mg^2+^. Thus, the exopolyphosphatase activity of PPN1 prevailed in the presence of Co^2+^, while the endopolyphosphatase activity prevailed in the presence of Mg^2+^. The endopolyphosphatase reaction was weak in the absence of bivalent cations (Fig. 2A).

We have studied the influence of some effectors on both activities in the presence of Mg^2+^ or Co^2+^ (Fig. 2). P_i_, PP_i_ and PolyP_3_ are the products of PolyP hydrolysis by PPN1 [19–21]. P_i_ had no effect on exopolyphosphatase [26] and endopolyphosphatase (Fig. 2B, C) activities. PP_i_ and PolyP_3_ inhibited P_i_ release (Fig. 2A) and PolyP chain shortening (Fig. 2 B, C) in the presence of both cations. The slight inhibitory effect of P_i_ at the concentrations above 5 mM and the inhibitory effect of PP_i_ on depolymerase activity of PPN1 in the presence of Mg^2+^ have been reported earlier [19, 20]. Heparin, an acid carbohydrate polymer, is a concurrent suppressor of exopolyphosphatases [26]. It also inhibited the endopolyphosphatase activity irrespective of the cation (Fig. 2B, C). Thus, there was no difference in the effects of the above compounds on the exopolyphosphatase and endopolyphosphatase activities of PPN1.

However, we have revealed that ATP, ADP and arginine differently affect the exo- and endopolyphosphatase activities of PPN1. Both P_i_ release and PolyP chain shortening in the presence of Co^2+^ were inhibited by ADP but not affected by ATP and arginine (Fig. 2 A, B). The P_i_ release in the presence of Mg^2+^ was not affected by ATP and arginine but slightly activated by ADP (Fig. 2A). The polyphosphate chain shortening in the presence of Mg^2+^ was activated by ADP and arginine but inhibited by ATP (Fig. 2C).

**Fig 3:**
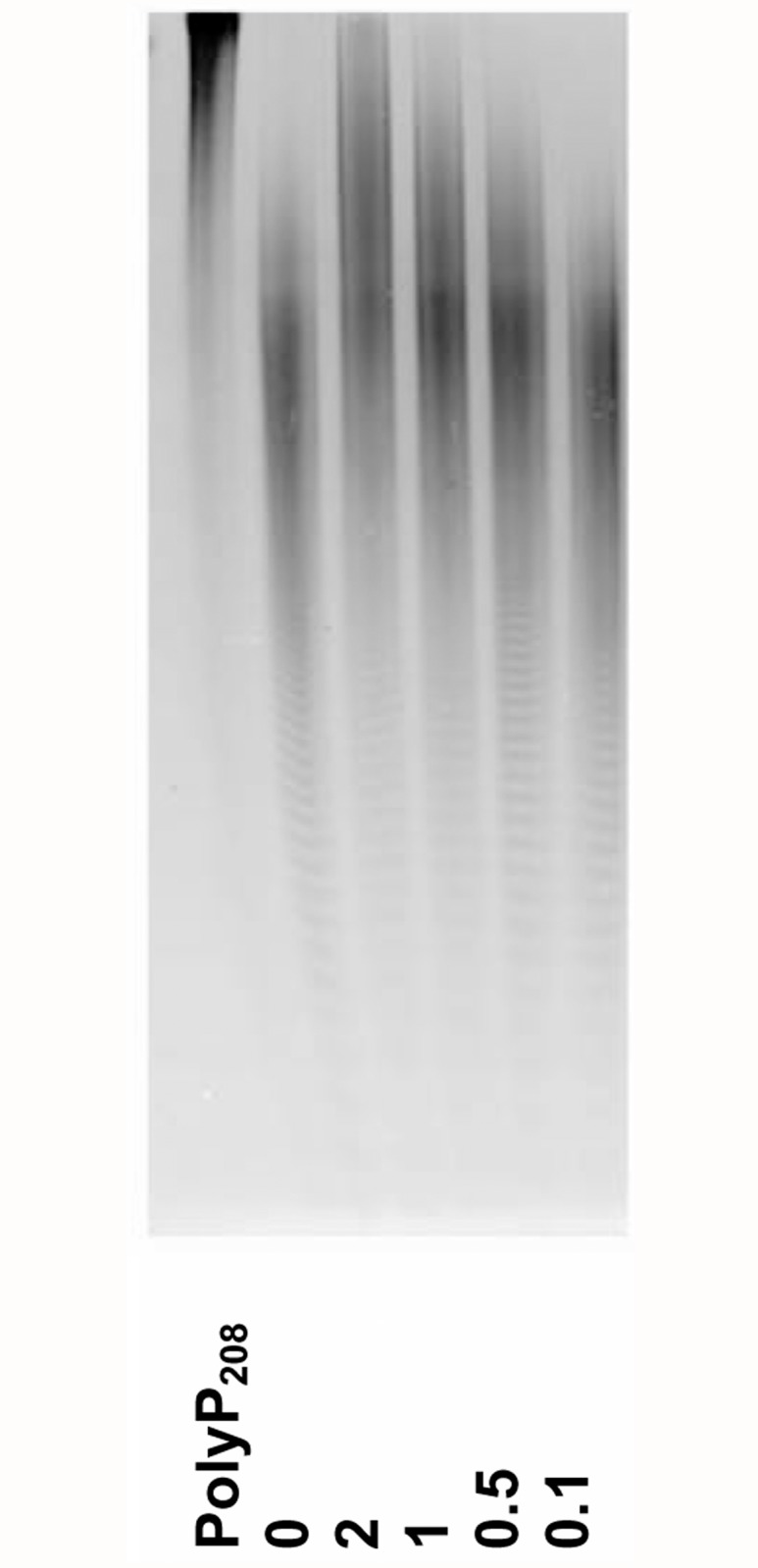
The effect of ATP on the endopolyphosphatase reaction of PPN1 in the presence of 0.25 mM MgSO_4_. PolyP PAGE was performed in 24% polyacrylamide gel with 7M urea; toluidine blue staining. PolyP_208_ - PolyP_208_ was incubated without the enzyme at 30°C for 60 min. The numerals indicate ATP concentrations (mM). The PAGE experiment was repeated in triple and the photograph of typical experiment is shown.

We have assessed the concentrations of ATP, ADP, arginine and amines, which influence the endopolyphosphatase activity in the presence of Mg^2+^. An example of such PAGE experiment is shown in Fig. 3: 0.5 mM ATP inhibited the endopolyphosphatase, while 0.1 mM was not effective. ADP stimulated the endopolyphosphatase even at 0.1 mM. Arginine stimulated the endopolyphosphatase activity at 50 mM but had no effect at 10 mM. In contrast to arginine, spermidine and 1,3-diaminopropane inhibited the endopolyphosphatase activity at 50 mM but were ineffective at 10 mM. At 50 mM, both polyamines inhibited the exopolyphosphatase activity independently of the bivalent cation (Fig. 2A).

## Discussion

We have revealed the conditions when PPN1 shows the prevalence of either exo- or endopolyphosphatase activity. The concentration of Mg^2+^ in yeast cytoplasm was reported to be ~1–5 mM [27]; the concentration of Co^2+^ was lower [28]. The Mg^2+^-dependent endopolyphosphatase activity of PPN1 seems to prevail in the cells of *S*. *cerevisiae* under common growth conditions. Yeasts are able to accumulate cobalt under its excess in the medium [28, 29]. Co^2+^ is localized mainly in vacuoles [29]. PPN1 is also present in vacuoles [18, 19]. We speculate the following way of Co^2+^ detoxification in vacuoles: PolyP degradation to P_i_ by PPN1 increases in the cells accumulating Co^2+^ and insoluble cobalt phosphate is formed.

The activities of many enzymes are regulated by ATP/ADP [30]. The intracellular concentrations of ATP and ADP in yeast depend on the growth stage and nutrient limitations [30–32]. ATP concentration varies from ~1 to 5 mM [30, 32]. ADP concentration was reported to be lower than 0.05 mM [33]. Thus, the physiological changes in ATP/ADP may be a factor of the PPN1 exo-/endopolyphosphatase switching. Depolymerization of PolyP was observed under glucose exhaustion during the growth of *S*. *cerevisiae* [34]. Probably, the decrease in ATP level and the increase in ADP level under these conditions stimulate PolyP depolymerization by PPN1.

The switching of exopolyphosphatase/endopolyphosphatase activity of PPN1 may be necessary for PolyP degradation depending on growth stage or under stress overcoming. PPN1 was found to be localized mainly in vacuoles and mitochondrial membranes. At the early stage of budding, the enzyme appears in the cytoplasm [26], where it seems to be responsible of PolyP fragmentation observed at a high yeast growth rate [35]. Its activity in the cytoplasm may be regulated by the ATP/ADP ratio.

PPN1 is present also in the vacuolar lumen [18, 36]. Under common growth conditions, pH value of the vacuolar lumen is acidic [37] and the enzyme with the neutral pH optimum [36] is inactive. Probably, the alkalization of vacuoles under stress caused by the excess of ammonium ions or heavy metal cations leads to an increase in the exopolyphosphatase activity; the enhanced hydrolysis of vacuolar PolyP to orthophosphate restores the pH value of the vacuoles. The degradation of NMR-visible, probably vacuolar, PolyP to short polymers in *S*. *cerevisiae* was observed under the conditions when it was necessary to neutralize the alkalization of cytoplasm [38]. The NMR study showed that the addition of 20 mM of NH_4_^+^ to *S*. *cerevisiae* cells caused a rapid increase in the cytoplasmic and vacuolar P_i_ and a breakdown of long-chain PolyP to short-chain PolyP and P_i_ [39]. Some part of the formed P_i_ is probably released from the vacuoles together with the toxic cations.

Arginine and amines also accumulate in the vacuoles; they are in complex with PolyP [37], and their ratio can also influence polyP degradation. The effective concentration of arginine in this study and the concentrations that activated the vacuolar exopolyphosphatases of *Neurospora crassa* [40] and *S*. *cerevisiae* [36] were similar. More detailed kinetic experiments are needed to reveal the cause of difference between the effects of arginine and amines.

Endopolyphosphatase activity seems to be essential in the yeasts possessing several enzymes, which fragment long chained PolyP into shorter ones. The endopolyphosphatase not encoded by the PPN1 gene was found in the double ΔPPX1/ΔPPN1 mutants [41]. In addition, the DDP1 protein exhibits an endopolyphosphatase activity [16]. The comparative analysis of the expression and regulation of these enzymes is of interest for understanding the role of PolyP in a yeast cell.

## Conclusions

The polyphosphatase PPN1 of *S*. *cerevisiae* possesses both endo- and exopolyphosphatase activities. The conditions for switching these activities have been revealed for the first time. In the presence of Mg^2+^, the enzyme shows a polyphosphate chain fragmentation activity, while Co^2+^ stimulates P_i_ release from the polyphosphate chain end. ATP inhibits while ADP activates endopolyphosphatase activity in the presence of Mg^2+^.
